# Effects of N_2_O elimination on the elimination of second gases in a two‐step mathematical model of heterogeneous gas exchange

**DOI:** 10.14814/phy2.15822

**Published:** 2023-11-03

**Authors:** Ben Korman, Ranjan K. Dash, Philip J. Peyton

**Affiliations:** ^1^ School of Medicine University of Western Australia Perth Western Australia Australia; ^2^ Department of Anaesthesia and Pain Medicine Royal Perth Hospital Perth Western Australia Australia; ^3^ Department of Biomedical Engineering Medical College of Wisconsin Milwaukee Wisconsin USA; ^4^ Department of Physiology Medical College of Wisconsin Milwaukee Wisconsin USA; ^5^ Anaesthesia, Perioperative and Pain Medicine Unit, Department of Anaesthesia, Austin Health, Melbourne Medical School University of Melbourne Heidelberg Victoria Australia

**Keywords:** anesthetic uptake and elimination, mathematical modeling, second gas effect, ventilation‐perfusion mismatch, volume contraction and expansion

## Abstract

We have investigated the elimination of inert gases in the lung during the elimination of nitrous oxide (N_2_O) using a two‐step mathematical model that allows the contribution from net gas volume expansion, which occurs in Step 2, to be separated from other factors. When a second inert gas is used in addition to N_2_O, the effect on that gas appears as an extra volume of the gas eliminated in association with the dilution produced by N_2_O washout in Step 2. We first considered the effect of elimination in a single gas‐exchanging unit under steady‐state conditions and then extended our analysis to a lung having a log‐normal distribution of ventilation and perfusion. A further increase in inert gas elimination was demonstrated with gases of low solubility in the presence of the increased ventilation‐perfusion mismatch that is known to occur during anesthesia. These effects are transient because N_2_O elimination depletes the input of that gas from mixed venous blood to the lung, thereby rapidly reducing the magnitude of the diluting action.

## Graphical abstract

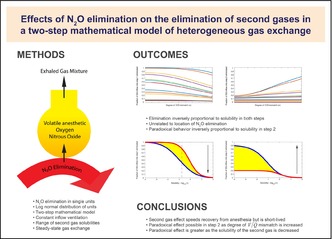

**New and Noteworthy**

Examination of gas transfer during nitrous oxide elimination using a two‐step mathematical model of steady‐state gas exchange suggests that unlike the situation during nitrous oxide uptake, the second gas effect is of a temporary nature. Paradoxical second gas effects are possible but will be short‐lived.

## 1 INTRODUCTION

Modern anesthetic practice aims to achieve safe, smooth induction and maintenance of anesthesia, followed by rapid recovery (Jakobsson & Davidson, 1999). Maintenance of anesthesia with inhalational agents remains popular, due to the ease and reliability of administration, and the ability to monitor inspired and alveolar anesthetic gas concentrations in real time. Nitrous oxide (N_2_O) has long been used due to its rapid onset and offset arising from its relatively low solubility in blood and tissues (blood/gas partition coefficient *λ* = 0.47), and its favorable side‐effect profile. Because of its low anesthetic potency, it is usual to combine high inspired concentrations of N_2_O with much lower concentrations of a more highly potent volatile anesthetic, which helps limit the cardiovascular and respiratory depressant effects of the volatile anesthetic if used alone, and its accumulation in body tissues (Patel & Goa, 1996). The *second gas effect* (SGE) occurs when a high inspired concentration of N_2_O (as “first gas”) is delivered during induction of anesthesia (Epstein et al., 1964; Stoelting & Eger, 1969). The resulting rapid uptake of large volumes of N_2_O, of the order of 0.5 L/min, from alveolar gas to pulmonary blood during the early stages of anesthesia (Korman & Mapleson, 1997; Peyton, Fortuin, et al., 2008), has a “concentrating” effect on other “second” gases present, increasing their alveolar partial pressures and accelerating their uptake.

At the end of surgery, the anesthetic gases are switched off, reversing the partial pressure gradients which now drive their elimination via the lungs. A SGE has been demonstrated in patients during this emergence stage of anesthesia, driven by the large volumes of N_2_O which move from the pulmonary circulation into alveolar gas producing a “diluting effect” (Peyton et al., 2011).

It has been shown that during induction and maintenance, the SGE is most powerful on second gas partial pressures in arterial blood, rather than alveolar gas, which has clinical importance given that their effect‐site concentrations in the brain are driven by delivered arterial partial pressures. This difference is due to ventilation‐perfusion scatter, which is significant in all anesthetized patients (Hendrickx et al., 2006; Korman et al., 2018; Peyton et al., 2001a, 2001b, 2001c, 2006; Peyton, Fortuin, et al., 2008; Peyton, Horriat, et al., 2008), because these perfusion‐driven concentrating effects of N_2_O uptake are most powerful in those lung compartments with relatively high blood flow and low $\dot{V}/\dot{Q}$ ratios. In fact, increasing $\dot{V}/\dot{Q}$ scatter in the lungs will paradoxically magnify the SGE. Furthermore, the SGE on blood partial pressures during anesthetic induction has been predicted to be greatest on SGs of lower solubility, such as the modern generation of volatile agents desflurane and sevoflurane, which further enhances its clinical importance.

Exactly how a low SG solubility combines with a high degree of $\dot{V}/\dot{Q}$ mismatch to paradoxically increase SG uptake in the SGE remained unexplained until the process of gas uptake in the lung was modeled as a two‐step process: gas exchange for all gases at constant volume followed by gas exchange on volume contraction with FG uptake (see Appendix 1) (Korman et al., 2020). The situation during the elimination of N_2_O was not examined and is therefore the subject of the current investigation. If present, a paradoxical SGE during the emergence phase of anesthesia would involve an increase in the predicted rate of gas elimination in the presence of an increase in the degree of $\dot{V}/\dot{Q}$ mismatch. Whether such a paradoxical effect is greater for low solubility second gases is also investigated.

## 2 METHODS

In order to study the effect of N_2_O elimination, we first describe gas elimination in a single gas‐exchanging unit of the lung under steady‐state conditions. We then extend the description to a nonhomogenous lung with a log normal distribution of $\dot{V}/\dot{Q}$ ratios characterizing the ventilation‐perfusion mismatch. Finally, the effect of volume expansion during N_2_O elimination is investigated using a two‐step mathematical model of steady‐state gas exchange. This follows the sequence of our previous work (Korman et al., 2020), now applied to the elimination phase of anesthesia.

### 2.1 Gas exchange in a single gas‐exchanging unit

Consider a gas with a linear dissociation curve characterized by a blood‐gas solubility coefficient *λ* being eliminated under steady‐state conditions. If such a gas is removed at a rate ${\dot{V}}_{A}F_{A}$ from a single gas‐exchanging unit of given ${\dot{V}}_{A}/\dot{Q}$, where ${\dot{V}}_{A}$ and $\dot{Q}$ are the expired alveolar gas and pulmonary capillary blood flowing from the unit, then the output of the gas is ${\dot{V}}_{A}F_{A}$, where *F*_A_ is the fractional concentration of the gas in the dry portion of the alveolar gas. This must be equal to the elimination by blood, which is given by $\lambda \dot{Q}\left(Fc^{\prime }-F\;\bar{v}\right)$, where $Fc^{\prime }$ and $F\;\bar{v}$ are given by $Pc^{\prime }/\left(P_{B}-P_{H_{2}O}\right)$ and $P\;\bar{v}/\left(P_{B}-P_{H_{2}O}\right)$, respectively, with $Pc^{\prime }$ and $P\;\bar{v}$ equal to the partial pressures of the gas in the alveolar end‐capillary blood and mixed venous blood, and $P_{B}$ and $P_{H_{2}O}$ are the barometric pressure and saturated vapor pressure of water at 37°C, respectively, so that:

(1) $$-{\dot{V}}_{A}F_{A}=\lambda \dot{Q}\left(Fc^{\prime }-F\;\bar{v}\right)$$

It is assumed here that the inspired gas mixture contains none of the gas being eliminated so that *F_I_* is zero. If the diluent gas is treated as if it were completely insoluble, we have

(2) $${\dot{V}}_{I}={\dot{V}}_{A}\left(1-F_{A}\right)$$

### 2.2 Constant inflow and constant outflow

We now consider the two extreme patterns of ventilation possible during steady‐state ventilation of a gas‐exchanging unit. With constant inflow, the inspired ventilation remains constant from breath to breath and the expired ventilation is allowed to vary. With constant outflow, it is the expired ventilation that remains constant from breath to breath and the inspired ventilation varies. These patterns were first described by Rackow et al. (1961) and are discussed in more detail elsewhere (Korman & Mapleson, 1997). Both patterns are possible during emergence from N_2_O anesthesia (Rackow et al., 1961), but the widespread use of constant volume ventilators in operating theaters suggests that the constant inflow pattern is most likely to be applicable. During expiration, constant volume ventilators are pressure generators, usually to atmospheric pressure, which conforms to the constant inflow pattern. Since constant outflow is not associated with any SGEs when the SG and N_2_O are eliminated simultaneously, (see Appendix 3), our focus in this paper is on constant inflow.

### 2.3 Constant inflow in a single gas‐exchanging unit

When ${\dot{V}}_{A}$ is eliminated from Equations (1) and (2), as required for the constant inflow case, we obtain the following expression for the alveolar and end‐capillary partial pressures of the FG (see Appendix 2):

(3) $$Fc^{\prime }=F_{A}=1-\frac{1}{2}\left\{\alpha +\sqrt{\alpha^{2}+4\psi }\right\}$$

where $\alpha =1-F\bar{v}-\psi$, $\psi ={\dot{V}}_{I}/\left(\lambda \dot{Q}\right)$, enabling ${\dot{V}}_{A}$ to be obtained from Equation (2). Equation (3) is used for N_2_O to determine ${\dot{V}}_{A}$ in each gas‐exchanging unit and the value of ${\dot{V}}_{A}$ then used in the following equation to calculate ${\dot{V}}_{A}F_{A}$, the outflow rate for the SG:

(4) $${\dot{V}}_{A}F_{A}=\frac{\lambda {\dot{V}}_{A}F\;\bar{v}}{\lambda +{\dot{V}}_{A}/\dot{Q}}$$

In Equation (4), λ and $F\;\bar{v}$ refer to the SG.

### 2.4 Extension to a nonhomogenous lung with a continuous distribution of $\dot{V}/\dot{Q}$ ratios

With a log normal distribution of $\dot{V}/\dot{Q}$ ratios, it is necessary to sum the flow‐weighted output of the gas‐exchanging units to determine the composition of mean alveolar gas and mixed pulmonary end‐capillary blood. The degree of mismatch between ventilation and blood flow is given by *σ*, the absolute value of the difference between the standard deviation (SD) of the logarithm of ventilation per unit volume and the SD of the logarithm of blood flow per unit volume (Colburn et al., 1974). In awake healthy adults, *σ* varies from 0.25 to 0.5; worsening of $\dot{V}/\dot{Q}$ matching is seen in disease states. It is also known to occur soon after induction of anesthesia with typical values of *σ* between 0.75 and 1.75 in healthy adults (Dueck et al., 1980; Lundh & Hedenstierna, 1983, 1984; Lundh & Johansson, 1983; Rehder et al., 1979). We set *σ* to appropriate values in order to mimic $\dot{V}/\dot{Q}$ mismatch during anesthesia. Alveolar ventilation ${\dot{V}}_{I}$ is set at 4 L/min, and pulmonary blood flow ${\dot{Q}}_{t}$ is set at 5 L/min.

### 2.5 Choice of second gas

To clarify the role of SG solubility, it is useful to select SGs with a wide range of solubilities in blood. We use a series of gases from sulfur hexafluoride (*λ* = 0.0076) to methoxyflurane (*λ* = 13) as SGs in our study of gas exchange at the alveolar level. For *λ* < 10, gas exchange occurs almost exclusively in the alveoli. For 10 < *λ* < 100, Anderson et al. have shown that there is a gradual transition from alveolar exchange to exchange in the conducting airways, while for *λ* > 100, gas exchange occurs almost completely in the airways (Anderson et al., 2003; Anderson & Hlastala, 2007). In addition, it is possible to treat gas exchange as a two‐step process (Korman et al., 2020). In Step 1, we calculate the component of gas transfer which would take place at constant volume. In Step 2, we determine the component of gas transfer which accompanies the subsequent correction in gas volume. This maneuver produces the same net transfer for each gas as the classical one‐step approach but allows us to study more closely the effect of net gas volume change. In each simulation, we assume that an inspired gas mixture containing 1% of the SG has previously been equilibrated with the body and is now being eliminated.

### 2.6 Nitrous oxide elimination

Since we are using N_2_O as a means of producing a volume change whose effects are to be studied, for convenience, we set *F_I_* to 0. $F{\bar{v}}_{N_{2}O}$ is selected so as to produce the required total expired alveolar ventilation, but not to exceed 0.7, the highest clinically effective concentration that can be used safely in the general population without producing hypoxia (Magnusson & Spahn, 2003). All programs were written in MATLAB. Further details of our technique are discussed elsewhere (Korman et al., 2018, 2019, 2020). The relationships used to generate the equations and figures are discussed further in Appendix 3.

## 3 RESULTS

**FIGURE 1:**
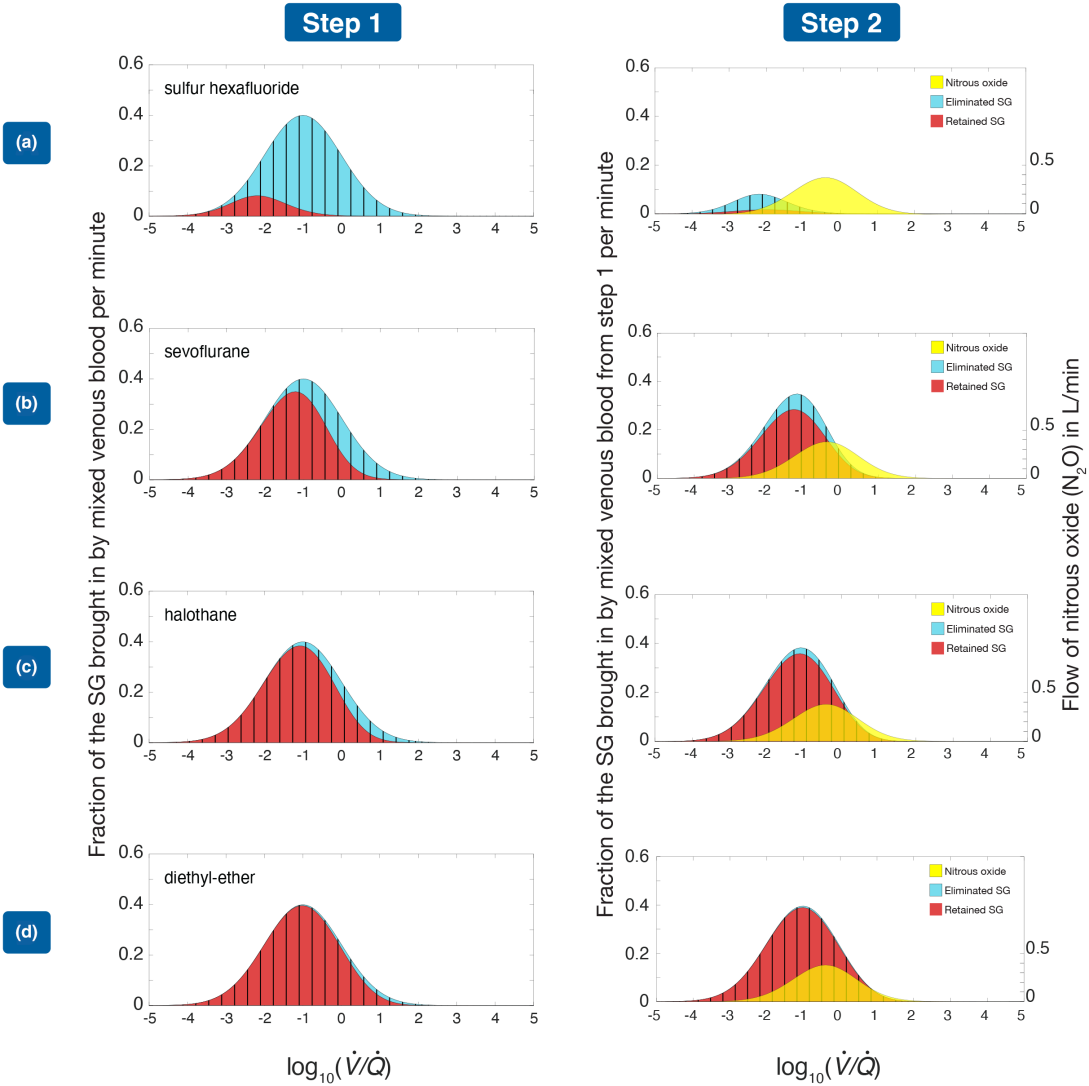
Elimination profile, with *σ* = 2, of sulfur hexafluoride (a), sevoflurane (b), halothane (c), and diethyl‐ether (d). Step 1: Distribution of each gas in inflowing mixed venous blood before (vertical‐hatched region) and after (red colored area) equilibration with alveolar gas but before N_2_O exchange is permitted. The distribution of the eliminated gas is shown in blue. Step 2: N_2_O elimination is now allowed to take place. The distribution of SG in pulmonary capillary blood at the end of Step 1 acts as the starting point for the further elimination in Step 2 (vertical‐hatched region in right‐hand panel) which accompanies the expansion in volume, producing the final distribution of SG in blood (red colored area). The distribution of the extra output is shown in blue. The yellow shading indicates the distribution of the eliminated N_2_O. Where this overlaps the SG, the yellow shading has been made partially transparent so as not to obscure the situation occurring with the SG.

In Figure 1, we have shown the situation for sulfur hexafluoride (*λ* = 0.0076), sevoflurane (*λ* = 0.59), halothane (*λ* = 2.3), and ether (*λ* = 12) — Step 1: elimination at constant volume, Step 2: elimination on the volume expansion associated with N_2_O elimination. The degree of $\dot{V}/\dot{Q}$ mismatch is high with *σ* = 2.

In Step 1, the vertical‐hatched region above the *x*‐axis indicates the SG brought in by the venous return. This includes both the red and blue sections. Since we have assumed a log normal distribution of blood flow, with the unit on the *x*‐axis being $\log_{10}\left(\dot{V}/\dot{Q}\right)$, the vertically hatched area in each case forms a bell‐shaped normal distribution. On the *y*‐axis, we have plotted the fraction of the incoming SG, so the vertically hatched area in each case equals 1. Following equilibration with alveolar gas, some of the SG is lost to the gas phase. The distribution of the lost gas is shown in blue. The remainder, whose distribution is shown in red, remains in pulmonary blood. The red region forms the output of Step 1, which then becomes the input to Step 2. In Step 2, the vertically hatched region again represents the distribution of the inflow from Step 1. It therefore has the same size, shape and location as the red area in Step 1. N_2_O transfer is now allowed to take place. Its distribution has been plotted in yellow and has been made somewhat transparent where it overlaps the result for the SG, so as not to obscure these other results. The unit used is L/min and is shown on the *y*‐axis on the right side of each box in Step 2. The eliminated N_2_O is associated with a further transfer of SG from pulmonary blood to alveolar gas, whose distribution is again shown in blue. As in Step 1, the distribution of the SG which remains in the blood phase is shown in red.

In each case, the amount of gas transferred in Step 2 is less than that transferred in Step 1; transfer in both steps behaves as expected being greatest with the least soluble gas sulfur hexafluoride and least with the most soluble gas, diethyl ether.

**FIGURE 2:**
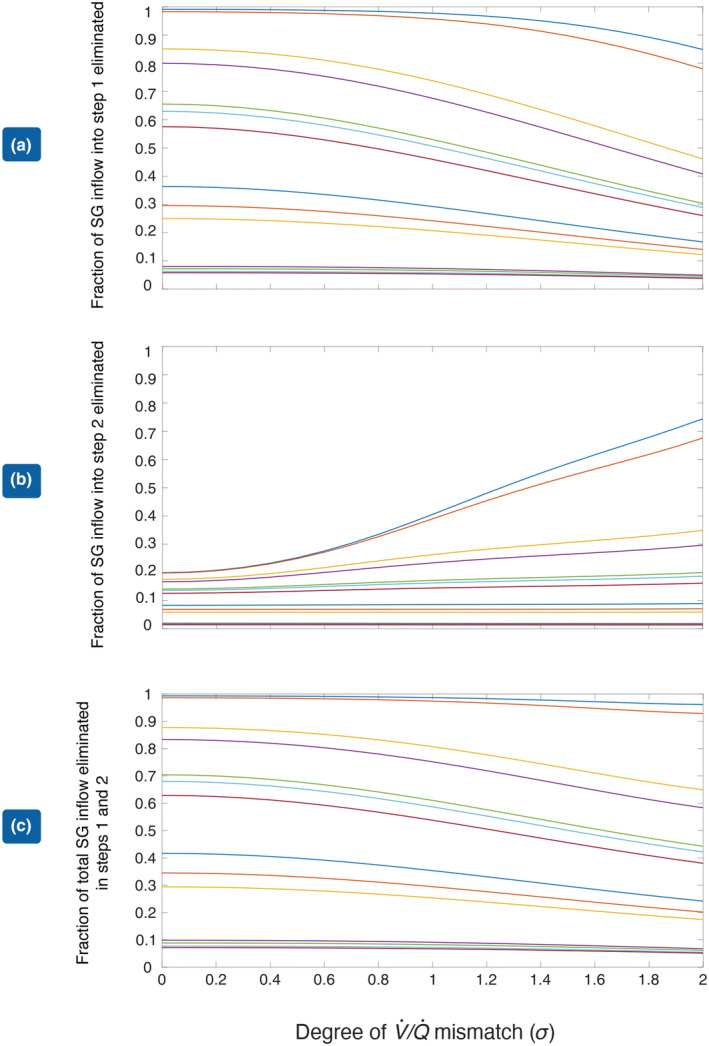
(a) Fraction of incoming SG eliminated in Step 1 as a function of *σ*, the degree of $\dot{V}/\dot{Q}$ mismatch. (b) Fraction of incoming SG eliminated in Step 2 as a function of *σ*. (c) Fraction of incoming SG eliminated in both steps as a function of *σ*, the degree of $\dot{V}/\dot{Q}$ mismatch Each line represents a different gas. From the top down: sulfur hexafluoride, nitrogen, ethylene, xenon, desflurane, sevoflurane, isoflurane, enflurane, halothane, trichloroethylene, chloroform, diethyl ether, and methoxyflurane.

In Figure 2, we show the effect of $\dot{V}/\dot{Q}$ mismatch on the elimination of various gases present in blood in low concentrations when N_2_O is eliminated from blood at a rate of 0.5 L/min. Figure 2a shows the effect in Step 1; Figure 2b shows the effect in Step 2; Figure 2c shows the total effect over the two steps. The least soluble gases appear at the top of each figure while the most soluble appear at the bottom.

In Figure 2a, the effect of increasing the degree of $\dot{V}/\dot{Q}$ mismatch is to decrease the transfer of the SG. This effect is most marked for the least soluble gases at the top of the diagram but minimal for the most soluble gases at the bottom. In Figure 2b, the egress of N_2_O is seen to increase the elimination of the SGs, as the degree of $\dot{V}/\dot{Q}$ mismatch increases, with the effect being maximal for the least soluble and minimal with the most soluble gases. The net effect shown in Figure 2c is to decrease the elimination of the SGs as the degree of mismatch increases but not to the same extent as is observed in the absence of N_2_O elimination (Step 1).

**FIGURE 3:**
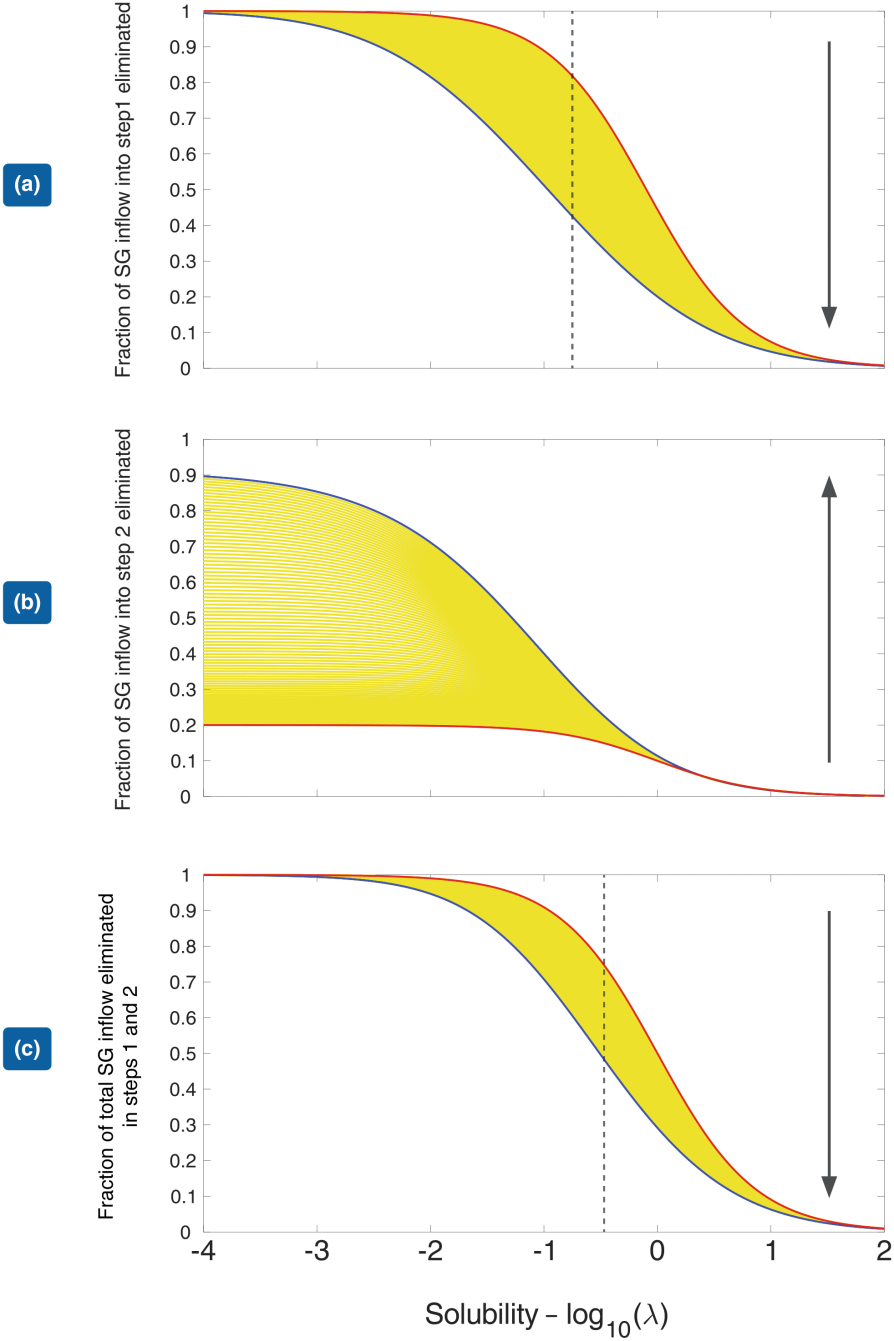
(a) Fraction of the incoming SG brought into Step 1 in blood, eliminated in Step 1 as a function of $\log_{10}\left(\lambda_{\mathrm{SG}}\right)$ for increasing degrees of $\dot{V}/\dot{Q}$ mismatch: red curve—no mismatch, blue curve—maximum mismatch. The yellow curves indicate the situation for increasing degrees of $\dot{V}/\dot{Q}$ mismatch between these two extremes. (b) Fraction of the incoming SG brought into Step 2 in blood (from Step 1), eliminated in Step 2 as a function of $\log_{10}\left(\lambda_{\mathrm{SG}}\right)$ for increasing degrees of $\dot{V}/\dot{Q}$ mismatch: red curve—no mismatch, blue curve—maximum mismatch. (c) Net elimination in Steps 1 and 2 as a fraction of the SG arriving in mixed venous blood expressed as a function of $\log_{10}\left(\lambda_{\mathrm{SG}}\right)$ for increasing degrees of $\dot{V}/\dot{Q}$ mismatch. The vertical black dashed line in Figure 3a,c gives the solubility for which there is maximum separation between the red and blue curves. The vertical black arrow shows the direction associated with an increase in $\dot{V}/\dot{Q}$ mismatch.

In Figure 3, we show the effect of solubility on the fraction of gas eliminated in each step (a and b) and on the overall transfer over both steps (c), for different degrees of $\dot{V}/\dot{Q}$ mismatch. Here, perfect matching between ventilation and blood flow, that is, *σ* = 0 is shown in red, the blue curve shows the situation with *σ* = 2, and the yellow region shows the situation for various values of *σ* between these two extremes. In Figure 3a, the red curve lies above the blue curve indicating that in the absence of the volume change which accompanies the elimination of N_2_O, the rate of SG transfer from blood to alveolar gas decreases as the degree of $\dot{V}/\dot{Q}$ mismatch increases. This is completely in accord with classical concepts of gas exchange. The difference between the two extreme cases is greatest when log_10_(*λ*) = −0.75, so *λ* = 0.2. This is shown by the dashed vertical black line. The difference diminishes at both ends of the solubility spectrum, virtually disappearing when *λ* > 10. In Figure 3b, we show the extra SG associated with the expansion of gas volume due to N_2_O elimination in Step 2. The position of the red and blue curves is reversed. Therefore during this step, SG elimination is enhanced as the degree of $\dot{V}/\dot{Q}$ mismatch is increased. The effect is greatest for the least soluble gases, but virtually disappears once *λ* > 1. This is in agreement with the result shown in Figure 2b above. Figure 3c shows the net effect of Steps 1 and 2. It can be seen that for any value of λ, the difference between the red and blue curves in Step 1 is reduced and the maximum difference has been shifted to the right and now occurs when log_10_(*λ*) = −0.5, that is, *λ* = 0.3 (dashed vertical black line).

## 4 DISCUSSION

For decades, the most‐widely accepted explanation of the SGE during induction of anesthesia relied solely on the contraction in gas volume associated with N_2_O uptake (Korman & Mapleson, 1997). The SGE was measured in expired gas and generally assumed to be of similar magnitude in blood. Since 2001, it has been shown that the effect is not only greater in blood, but also continues to be significant for sevoflurane and desflurane when N_2_O uptake falls to relatively low steady‐state levels and that this is due to the mismatch between ventilation and blood flow that occurs routinely following the induction of anesthesia (Hendrickx et al., 2006; Korman et al., 2018; Peyton et al., 2001a, 2001b, 2001c, 2006; Peyton, Fortuin, et al., 2008; Peyton, Horriat, et al., 2008). According to basic concepts of respiratory physiology, $\dot{V}/\dot{Q}$ mismatch should have the opposite effect on gas transfer (Neufeld et al., 1978; West et al., 1974; West & Wagner, 1977). This remained unexplained until it was shown that the process of gas uptake in the lung may be treated theoretically as a two‐step process: gas exchange at constant volume followed by gas exchange on volume contraction (Korman et al., 2020). This is achieved by only allowing the SG to equilibrate with blood in the Step 1. Since it is present in very low concentrations, the associated volume change is negligible. Gas uptake is completed in Step 2, in which the first gas is allowed to equilibrate with blood producing a contraction in volume which results in an extra uptake of SG, as outlined in detail in Appendix 1.

We have applied similar techniques to the elimination of a SG during N_2_O washout as previously used by us in relation to the uptake of SGs during N_2_O washin (Korman et al., 2020). Paradoxical behavior is demonstrable in Step 2 as evidenced in Figures 2b and 3b. In the case of gas uptake, we have previously shown how $\dot{V}/\dot{Q}$ mismatch can increase gas transfer from gas phase to blood. The primary mechanism involves a contraction in gas volume, which accompanies N_2_O uptake producing an increase in the SG partial pressure gradient, which results in a further uptake of the SG. The increase in SG partial pressure gradient is most effective if the loss of SG in Step 1 is minimized, which depends on having a gas with a low solubility in blood and increases as the degree of $\dot{V}/\dot{Q}$ mismatch increases. Components of this mechanism may be identified during the elimination phase of anesthesia.

From Figure 2a, it can be seen that gases with low solubility in blood are most sensitive to an increase in the degree of $\dot{V}/\dot{Q}$ mismatch. As a result, the fraction of SG in mixed venous blood transferred to alveolar gas in Step 1 falls as the degree of $\dot{V}/\dot{Q}$ mismatch increases, whereas such increases have virtually no effect on the fraction of soluble gases such as ether and methoxyflurane eliminated in Step 1. The egress of N_2_O from blood to alveolar gas now dilutes the SG, lowering its partial pressure and increasing the partial pressure gradient for the SG across the alveolar‐capillary membrane. In turn, this produces the extra gas transfer shown in the right‐hand column of Figure 1. This, then, is the SGE during N_2_O elimination. The paradoxical effect of $\dot{V}/\dot{Q}$ mismatch on this additional elimination in Step 2, apparent for gases with a low solubility in blood, may be explained by their proportionately greater retention in blood as *σ* is increased. This is shown clearly in Figure 2a, in which the upper curves representing less soluble gases all slope downwards as the degree of $\dot{V}/\dot{Q}$ mismatch is increased.

A second contributing factor is the location of gas exchange in the $\dot{V}/\dot{Q}$ spectrum for each gas and how the location is affected as the $\dot{V}/\dot{Q}$ mismatch is increased. As shown in our previous study, this is significant during induction and maintenance of anesthesia for highly soluble gases whose remainder after Step 1 is not aligned with the contraction in volume which occurs in Step 2 (Korman et al., 2020). In the case of diethyl‐ether, Figure 1 shows excellent overlap in Step 2, between the residual gas (red area) and the volume expansion associated with N_2_O elimination (yellow area). In spite of this, ether is so tightly held in blood that very little is eliminated in Step 2. These findings are in accord with the slower recovery times associated with the use of this gas.

Although its contribution during induction is small, accounting for at most a 10% increase in the rate of gas uptake during the early stages of N_2_O washin, the SGE has attracted a large amount of attention. Two important questions arise: does it exist, is it significant (Lee & Sun, 1999; Mapleson & Korman, 1999)? The demonstration of a persistent SGE with sevoflurane during anesthesia maintenance has recently generated fresh interest, primarily because of the association with an increase in $\dot{V}/\dot{Q}$ mismatch, known to occur during anesthesia. During induction, increases in blood partial pressures as great as 25% may occur (Peyton, Horriat, et al., 2008). There are, however, important differences between emergence and the induction/maintenance phase of anesthesia which act to reduce the significance of any SGEs demonstrated here. During induction of anesthesia the anesthesiologist will commonly hasten washin of a volatile anesthetic agent for a few minutes using “overpressure,” the deliberate delivery of a high fresh gas flow and high inspired concentration, which produces a greater effect on the partial pressure of the SG in alveolar gas and pulmonary blood than the SGE alone. Prior to the elimination phase, the inflow of N_2_O is maintained relatively constant via the fresh gas mixture being delivered to the breathing circuit. This acts as a constant driver for the SGE. However, during emergence, following replacement of anesthetic gas delivery to the patient with oxygen, there is little available to the anesthesiologist to accelerate emergence from inhalational anesthesia. The contribution of N_2_O washout to emergence is therefore beneficial but because of its low solubility in blood and tissues, body stores of N_2_O are rapidly depleted, so that the input to the lungs from the flow of mixed venous blood rapidly declines. The volume expansion phase is therefore expected to be short‐lived, thereby limiting the magnitude and duration of any SGEs. Furthermore, the $\dot{V}/\dot{Q}$ mismatch responsible for the paradoxical effects both during N_2_O uptake and elimination disappears shortly after the cessation of anesthesia, so, unlike the situation during maintenance, these effects will not be expected to persist for long. Masuda and Ikeda demonstrated a 18% reduction in end‐tidal halothane concentrations on cessation of N_2_O due to the SGE (Masuda, 1984). Peyton et al. (2011) obtained a 37% reduction in arterial sevoflurane concentrations 2 min after cessation of N_2_O with a 22% reduction simultaneously in end‐tidal gas. These results are in keeping with a paradoxical SGE but the results were not statistically significant. The significant features of the SGE during N_2_O uptake and elimination are shown in Table 1.

**TABLE 1:** Features of second gas transfer during uptake and elimination phases of anesthesia.

Direction of N_2_O transfer	Uptake	Elimination
SGE with constant outflow	Significant	Absent
SGE with constant inflow	Reduced c.f. constant outflow	Present
Nature of volume change	Contraction	Expansion
Mechanism of SGE production	Concentrating effect	Diluting effect
Role of SG solubility	Effect maximal with λ≈0.5	SGE inversely proportional to solubility
Role of $\dot{V}/\dot{Q}$ mismatch	Decreases SGE in gas phase, increases SGE in blood	No effect on transfer of highly soluble gases; impedes transfer of less soluble gases
Paradoxical SGE with $\dot{V}/\dot{Q}$ mismatch	Predicted theoretically and demonstrated experimentally	Possible but far less likely with sevoflurane and desflurane. Difficult to measure
Duration of SGE	Persists for long periods	Short‐lived
Importance of N_2_O overlap as in Figure 1	Important	Unimportant

The question of whether the two‐step mathematical analysis is a valid representation of events in the lung, has been raised in discussion with the authors. Our view is that given the new insights it has yielded into gas exchange in the lung, it should be regarded in the same way as the vector representation of motion in 2 or 3 dimensions—a useful tool which yields accurate results and provides deeper understanding of the underlying processes. Certainly, the increase in the SGE which accompanies an increase in $\dot{V}/\dot{Q}$ mismatch during induction of anesthesia with N_2_O is not easily explained in any other way.

## 5 CONCLUSION

We have investigated the elimination of inert gases during the elimination of N_2_O in the lung using a two‐step mathematical model, that allows the contribution from net gas volume expansion, which occurs in Step 2, to be separated from other factors. When both gases are being eliminated simultaneously, the SGE appears as an extra volume of gas eliminated in association with the dilution produced by N_2_O washout. A paradoxical increase in SGE also occurs with agents of low solubility in the presence of $\dot{V}/\dot{Q}$ mismatch. Both the SGE and the paradoxical effect of $\dot{V}/\dot{Q}$ mismatch are expected to be short‐lived because N_2_O elimination depletes the input of that gas from mixed venous blood to the lung, thereby rapidly reducing the magnitude of the diluting action of N_2_O.

## FUNDING INFORMATION

HHS|National Institutes of Health (NIH): Ranjan K. Dash, U01‐HL122199; HHS|National Institutes of Health (NIH): Ranjan K. Dash, P01‐GM066730.

## ETHICS STATEMENT

There are no ethical issues here. The work is ours completely and does not involve any experiments on living creatures.

## Data Availability

This is not relevant. We have provided the equations and described the techniques involved in generating our results. Any interested reader is free to use these techniques for their own simulations.

## APPENDIX 1

### Mathematical basis for the two‐step treatment of gas exchange in the lung

We proceed first by obtaining a result for the transfer of gas in a single gas‐exchanging unit of the lung under steady‐state conditions. This result is then extended to the non‐steady state and finally, to the lung as a whole. Although we use an inert gas in our derivation, it is shown that the result is independent of the relationship governing the interaction of the gas with blood and is therefore general in nature.

Consider an inert gas being exchanged under steady‐state conditions in a single gas‐exchanging unit of given ${\dot{V}}_{A}/\dot{Q}$ in the lung. If such a gas is delivered in the inspired gas mixture at a rate ${\dot{V}}_{I}F_{I}$, (the *inflow* of the gas), where ${\dot{V}}_{I}$ is the inspired ventilation of the unit in L/min and $F_{I}$ is the fractional concentration of the gas in the inspired gas mixture and removed at a rate ${\dot{V}}_{A}F_{A}$, (the *outflow* of the gas), where ${\dot{V}}_{A}$ is the expired ventilation of the unit in L/min and $F_{A}$ is the fractional concentration of the gas in the expired gas mixture, then the uptake of the gas is ${\dot{V}}_{I}F_{I}-{\dot{V}}_{A}F_{A}$. (If the gas is being eliminated from blood, ${\dot{V}}_{I}F_{I}-{\dot{V}}_{A}F_{A}$ will be negative.) This must equal the uptake by blood which is given by $\lambda \dot{Q}\left(Fc^{\prime }-F\bar{v}\right)$, where *λ* is the blood/gas partition coefficient of the gas at 37°C, and $\dot{Q}$ is the blood flow to the unit. Here, $Fc^{\prime }$ and $F\;\bar{v}$ are equal to $Pc^{\prime }/\left(P_{B}-P_{H_{2}O}\right)$ and $P\bar{v}/\left(P_{B}-P_{H_{2}O}\right)$ respectively, with $Fc^{\prime }$ the fractional concentration in the dry fraction of a gas sample in equilibrium with the capillary blood draining the unit and $Pc^{\prime }$ the partial pressure of that gas, so that we have the following mass‐balance relationship for the gas:

(A1)
$${\dot{V}}_{I}F_{I}-{\dot{V}}_{A}F_{A}=\lambda \dot{Q}\left(Fc^{\prime }-F\;\bar{v}\right)$$

In the case of the physiological gases O_2_ and CO_2_, the expression on the right‐hand side of this equation is different because of the complicated binding with hemoglobin. Either side of Equation (A1) may be taken as the rate of transfer of gas. Let us therefore denote the rate of transfer of some gas G by the symbol ${\dot{V}}_{G}$. We may then write:

(A2)
$${\dot{V}}_{G}={\dot{V}}_{I}F_{I}-{\dot{V}}_{A}F_{A}$$

Consider now this process as taking place in two steps, the first step at constant volume ${\dot{V}}_{I}$, the second step during the volume correction from ${\dot{V}}_{I}$ to ${\dot{V}}_{A}$. If we denote events during step one as “(01),” those during Step 2 as “(12),” and those over both steps as “(02),” the two‐step assumption gives rise to the following pair of equations:

(A3)
$${\dot{V}}_{G}\left(01\right)={\dot{V}}_{I}F_{I}-{\dot{V}}_{I}F^{\prime }$$

(A4)
$${\dot{V}}_{G}\left(12\right)={\dot{V}}_{I}F^{\prime }-{\dot{V}}_{A}F_{A}$$

In Equation (A3), $F_{I}$ and $F^{\prime }$ are the fractional concentrations in dry gas at the start and finish of Step 1 and ${\dot{V}}_{G}\left(01\right)$ is the rate of uptake of the gas in this step (or the rate of elimination). Similarly, in Equation (A4), $F^{\prime }$ and $F_{A}$ are the fractional concentrations in dry gas at the start and finish of Step 2 and ${\dot{V}}_{G}\left(12\right)$ is the uptake (or output) of the gas in this step. It follows from the addition of Equations (A3) and (A4) by comparison with Equation (A2) that ${\dot{V}}_{G}={\dot{V}}_{G}\left(01\right)+{\dot{V}}_{G}\left(12\right)$ and so may also be denoted by the symbol ${\dot{V}}_{G}\left(02\right)$. Furthermore, if we denote the rate of N_2_O elimination in Step 2 as ${\dot{V}}_{N_{2}O}$, we can write ${\dot{V}}_{A}={\dot{V}}_{I}+{\dot{V}}_{N_{2}O}$, and ${\dot{V}}_{A}\left(02\right)={\dot{V}}_{A}\left(01\right)+{\dot{V}}_{A}\left(12\right)$, where ${\dot{V}}_{A}\left(01\right)={\dot{V}}_{I}$ and ${\dot{V}}_{A}\left(12\right)={\dot{V}}_{N_{2}O}$.

Note that the output of Equation (A3), ${\dot{V}}_{I}F^{\prime }$, becomes the input to Equation (A4), that is, the output of Step 1 becomes the input to Step 2. Moreover, this treatment makes no assumptions about the nature of the relationship governing the interaction between the gas G and blood and is therefore always applicable to gas exchange under steady‐state conditions. In fact, Rahn and Fenn used this property in a graphical derivation of the alveolar gas equation (Rahn & Fenn, 1955). Furthermore, if we add the term $d\left({\mathrm{VF}}_{A}\right)/dt$ to the right‐hand side of Equation (A1), where *V* is the volume of this individual gas‐exchanging unit, we obtain the following equation which now includes the extra term to allow for the change in the amount of the gas in the single gas‐exchanging unit as a function of time:

(A5)
$${\dot{V}}_{I}F_{I}-{\dot{V}}_{A}F_{A}=\lambda \dot{Q}\left(Fc^{\prime }-F\;\bar{v}\right)+d\left({\mathrm{VF}}_{A}\right)/dt$$

This maneuver allows us to extend the property described in Equations (A3) and (A4) to the non‐steady state, so that it becomes a *fundamental property of gas exchange in individual gas exchanging units*. Another way of stating this property is that it is always possible to find $F^{\prime }$ such that Equations (A3) and (A4) are satisfied. By summing over all gas‐exchanging units, the property may be extended to gas exchange in the lung as a whole. To apply this theory, we proceed as follows.

In the case of an inert SG being eliminated together with N_2_O under steady‐state conditions, as postulated in this article, we note that $F_{I}$ is zero for both N_2_O and the SG. Equation (A1) then becomes:

(A6)
$${\dot{V}}_{A}F_{A}=\lambda \dot{Q}\left(F\;\bar{v}-Fc^{\prime }\right)$$

We deal first with N_2_O, obtaining values for ${\dot{V}}_{A}$ and $F_{A}$ by solving Equations (2) and (A6) simultaneously. As we have specified the constant inflow case, ${\dot{V}}_{I}$ is known, and may be substituted in Equation (2). For inert gases, equilibration between gas and blood is complete, so $Fc^{\prime }=F_{A}$. The other variables in Equation (A6) are known.

With ${\dot{V}}_{A}$ known, we now turn our attention to the SG. Equation (A6) is solved to obtain $F_{A}$ for the SG and ${\dot{V}}_{G}\left(02\right)$ calculated from the product of ${\dot{V}}_{A}F_{A}$. We next deal with Step 1 for the SG, in which gas elimination occurs at constant volume, so we set ${\dot{V}}_{A}={\dot{V}}_{I}$. With ${\dot{V}}_{A}$, $Fc^{\prime }$, and $F\;\bar{v}$ known, Equation (A6) may now be solved for $F_{A}$ to provide the value of $F^{\prime }$ and the associated value of ${\dot{V}}_{G}\left(01\right)={\dot{V}}_{I}F^{\prime }$. ${\dot{V}}_{G}\left(12\right)$ may now be obtained by subtracting ${\dot{V}}_{G}\left(01\right)$ from ${\dot{V}}_{G}\left(02\right)$.

To extend the process to the lung as a whole, a specific distribution must be assumed for ventilation and blood flow as a function of $\log\left(\dot{V}/\dot{Q}\right)$. We use a log normal distribution of $\dot{V}/\dot{Q}$ ratios (see Appendix 3 below). The above calculations may then be performed for each compartment and the total gas eliminated in each step obtained by summing the values for each compartment. This is achieved most readily by using vector algebra as illustrated in Appendix 3.

## APPENDIX 2

### Derivation of Equation (3)

Assuming equilibration so that $Fc^{\prime }=F_{A}$, from Equations (1) and (2) we obtain,

(A7)
$$\lambda \dot{Q}F\bar{v}-\lambda \dot{Q}F_{A}=\frac{{\dot{V}}_{I}F_{A}}{\left(1-F_{A}\right)}$$

where the symbols $\lambda ,F\bar{v},F_{A}$ refer to N_2_O. Using the convenient substitution $w=1-F_{A}$, we obtain:

(A8)
$$\lambda \dot{Q}F\bar{v}-\lambda \dot{Q}\left(1-w\right)=\frac{{\dot{V}}_{I}\left(1-w\right)}{w}$$

On multiplying through, Equation (A8) becomes:

(A9)
$$\lambda \dot{Q}w^{2}-\lambda \dot{Q}\left(1-F\bar{v}\right)w+w{\dot{V}}_{I}+{\dot{V}}_{I}=0$$

Dividing by $\lambda \dot{Q}$ and setting $\psi ={\dot{V}}_{I}/\left(\lambda \dot{Q}\right)$ produces the following quadratic equation:

(A10)
$$w^{2}-\left(1-F\bar{v}-\psi \right)w-\psi =0$$

With solutions $w=0.5\left\{\left(1-F\bar{v}-\psi \right)\pm \sqrt{{\left(1-F\;\bar{v}-\psi \right)}^{2}+4\psi }\right\}$. Since $0\leq F_{A}\leq 1$, ω must also lie between these limits so only the positive root applies. Then, $F_{A}=1-w$.

## APPENDIX 3

### The Colburn equations

The probability density functions for ventilation $\left(\dot{V}\right)$ and blood flow $\left(\dot{Q}\right)$ derived by Colburn et al. (1974) are as follows:

(A11)
$$\dot{V}=\frac{{\dot{V}}_{A}}{\sqrt{2\pi }}e^{-x^{2}/2}$$

(A12)
$$\dot{Q}=\frac{{\dot{Q}}_{t}}{\sqrt{2\pi }}e^{-{\left(x-\sigma \right)}^{2}/2}$$

Here, ${\dot{V}}_{A}$ and ${\dot{Q}}_{t}$ are the total expired alveolar ventilation and total pulmonary blood flow, respectively, and *σ* is as previously defined. Defining $v=\dot{V}/{\dot{V}}_{A}$ as the fractional ventilation and $q=\dot{Q}/{\dot{Q}}_{t}$ as the fractional blood flow, Equation (A12) may be solved for *x* giving:

(A13)
$$\;x=\frac{\sigma }{2}-\frac{\log\left(v/q\right)}{\sigma }$$

Using $\left(1/\sigma \right)\log\left(v/q\right)$ as the independent variable in Equations (A11) and (A12), and letting μ=σ/2, we obtain the following useful equations for $\dot{V}$ and $\dot{Q}$:

(A14)
$$\dot{V}=\frac{{\dot{V}}_{A}}{\sqrt{2\pi }}e^{-{\left(x-\mu \right)}^{2}/2}$$

(A15)
$$\dot{Q}=\frac{{\dot{Q}}_{t}}{\sqrt{2\pi }}e^{-{\left(x+\mu \right)}^{2}/2}$$

With constant inflow, we substitute ${\dot{V}}_{I}$ (equal to 4 L/min) for ${\dot{V}}_{A}$ in these equations.

### Derivation of distributions of inflow, elimination, retention, and outflow for use in diagrams

We start by defining the interval on the *x*‐axis to be partitioned into *n* equal intervals. Since we have specified that $F_{I}$ equals zero for each gas under consideration, the only input we need to consider is gas being brought into the lung by the pulmonary circulation. Therefore, an acceptable interval is where $\left(-\mu -3\right)\leq x\leq \left(\mu +3\right)$. If we restrict *σ* to the interval [0, 2], a partition on the interval [−5, 5] can be used throughout. Choosing *n* = 1000 will then provide 101 points in each unit sub‐interval, for example, from −5 to −4, each of the *n* intervals will be 0.01 units wide. This will generally provide sufficient coverage. We now have a partition $\left\{x_{k}\right\}$ of the interval [−5, 5] with *n* + 1 points such that $-5<x_{1}<x_{2}\cdots \cdots x_{n}<x_{n+1}<5$ and define the vector $x=\left[x_{1}\cdots \cdots \cdots x_{n+1}\right]$. We next distinguish the different stages of gas uptake as follows:

0 = initial introduction of the gas G under consideration.
1 = situation at the end of Step 1.
2 = situation at the end of Step 2.

The change in any parameter in Step 1 is then indicated by the abbreviation (01) after the symbol of that parameter and that in Step 2 by the abbreviation (12). The total change in Steps 1 and 2 is indicated by the abbreviation (02).

For each $x_{k}$ in x, we define the following parameters and the associated vectors where *λ* and $F\bar{v}$ refer to the gas G:

The inspired alveolar gas flow:

(A16)
$$\begin{array}{l} {\dot{V}}_{I_{k}}=\left\{\frac{{\dot{V}}_{I}}{\sqrt{2\pi }}e^{-{\left(x_{k}-\mu \right)}^{2}/2}\right\}\\ {\dot{V}}_{I}=\left[{\dot{V}}_{I_{1}}.........{\dot{V}}_{I_{n+1}}\right] \end{array}$$

(A17)
$$\begin{array}{l} {\dot{V}}_{A_{k}}={\dot{V}}_{I}/\omega_{k}\\ \omega_{k}=0.5\left(\alpha_{k}+\sqrt{\alpha_{k}^{2}+4\psi_{k}}\right)\\ \psi_{k}={\dot{V}}_{I_{k}}/\left(\lambda {\dot{Q}}_{k}\right)\\ \alpha_{k}=1-\psi_{k}-\mathrm{Fv}\\ {\dot{V}}_{A}=\left[{\dot{V}}_{A_{1}}.........{\dot{V}}_{A_{k+1}}\right] \end{array}$$

The expired alveolar gas flow:

(A18)
$$\begin{array}{l} {\dot{Q}}_{k}=\frac{{\dot{Q}}_{t}}{\sqrt{2\pi }}e^{-{\left(x_{k}+\mu \right)}^{2}/2}\\ \dot{Q}=\left[{\dot{Q}}_{1}.........{\dot{Q}}_{n+1}\right] \end{array}$$

The pulmonary capillary blood flow:

(A19)
$$\begin{array}{c} \begin{array}{l} {\dot{Q}}_{G}{\left(01\right)}_{k}=\frac{\lambda {\dot{V}}_{I_{k}}F\bar{v}}{\lambda +\left({\dot{V}}_{I_{k}}/{\dot{Q}}_{k}\right)}\\ {\dot{Q}}_{G}\left(01\right)=\left[{\dot{Q}}_{G}{\left(01\right)}_{1}.........{\dot{Q}}_{G}{\left(01\right)}_{n+1}\right] \end{array} \end{array}$$

Elimination of gas G from blood in Step 1:

(A20)
$$\begin{array}{c} \begin{array}{l} {\dot{Q}}_{G}\left(02\right)=\;\frac{\lambda {\dot{V}}_{A}F\bar{v}}{\lambda +\left({\dot{V}}_{A}/{\dot{Q}}_{t}\right)}\\ {\dot{Q}}_{G}\left(02\right)=\left[{\dot{Q}}_{G}{\left(02\right)}_{1}.........{\dot{Q}}_{G}{\left(02\right)}_{n+1}\right] \end{array} \end{array}$$

Combined uptake of gas G in Steps 1 and 2:

The inflow of gas G in Step 1 is obtained by multiplying the vector $\dot{Q}$ by $\mathrm{\lambda F}\bar{v}$. The gas retained at the end of Step 1 becomes the outflow of the gas G at the end of Step 1. This in turn becomes the inflow of gas G in Step 2. The uptake of gas G in Step 2 is given by ${\dot{Q}}_{G}\left(12\right)={\dot{Q}}_{G}\left(02\right)-{\dot{Q}}_{G}\left(01\right)$. By using vector algebra, it is possible to obtain the distribution of any required parameter. The total outflow of gas over the whole range of *x* values in each step may be obtained by applying the *trapezoidal rule* (Karris, 2007).

### Constant outflow with both gases being eliminated simultaneously

The simplest explanation of why there can be no SGE with constant outflow is based on the properties of ideal gas mixtures. Thus, because we are operating at atmospheric pressure, mixtures of N_2_O and SG are considered “ideal” or “perfect” in the thermodynamic sense, that is, they obey Boyle's Law, Charles' Law, and the Ideal Gas Equation (*PV* = *nRT*). There are no interactions between the molecules of different species in such mixtures so that SG molecules cannot “see” the N_2_O molecules and vice versa (Denbigh, 1981). The only way for the N_2_O, flowing out of pulmonary blood, to influence the elimination of any SG is by increasing ${\dot{V}}_{A}$, which in turn, provides a greater volume of gas into which the SG can diffuse. With constant outflow, this gas volume remains constant, thus preventing N_2_O from having any effect on the elimination of the SG. This point has also been made by Mapleson (1964). If, instead of being eliminated at the same time as the N_2_O, we have a scenario during anesthesia, in which a N_2_O/O_2_ inspired gas mixture is replaced by an O_2_/SG mixture under constant outflow conditions, the elimination of N_2_O can impede the washin of the SG‐containing mixture by reducing ${\dot{V}}_{I}$ (Mapleson, 1963). It follows, that if there is a diluting effect during the elimination of N_2_O, as demonstrated theoretically in this paper and observed experimentally elsewhere (Peyton et al., 2011), some component of constant inflow must be present.
